# Efficacy of Dry Heat Treatment against *Clostridioides difficile* Spores and *Mycobacterium tuberculosis* on Filtering Facepiece Respirators

**DOI:** 10.3390/pathogens11080871

**Published:** 2022-08-02

**Authors:** Aswathi Soni, Natalie A. Parlane, Farina Khan, José G. B. Derraik, Cervantée E. K. Wild, Yvonne C. Anderson, Gale Brightwell

**Affiliations:** 1 Food System Integrity, Hopkirk Research Institute, AgResearch, Palmerston North 4442, New Zealand; aswathi.soni@agresearch.co.nz; 2 Animal Health Solutions, Grasslands Research Centre, Hopkirk Research Institute, AgResearch, Private Bag 11008, Palmerston North 4442, New Zealand; natalie.parlane@agresearch.co.nz (N.A.P.); farina.khan@agresearch.co.nz (F.K.); 3 Department of Pediatrics: Child and Youth Health, University of Auckland, Private Bag 92109, Auckland 1142, New Zealand; j.derraik@auckland.ac.nz (J.G.B.D.); cervantee.wild@auckland.ac.nz (C.E.K.W.); 4 Nuffield Department of Primary Care Health Sciences, University of Oxford, Oxford OX2 6GG, UK; 5 enAble Institute, Faculty of Health Sciences, Curtin University, Bentley, WA 6102, Australia; 6 Telethon Kids Institute, Northern Entrance, Perth Children’s Hospital, Nedlands, WA 6009, Australia; 7 Community Health, Child and Adolescent Health Service, Perth, WA 6009, Australia; 8 New Zealand Food Safety Science Research Centre, Palmerston North 4474, New Zealand

**Keywords:** bacteria, COVID-19, deionized water, disinfection, healthcare workers, inactivation, personal protective equipment, PPE, reuse, SARS-CoV-2, soil load

## Abstract

The COVID-19 pandemic has required novel solutions, including heat disinfection of personal protective equipment (PPE) for potential reuse to ensure availability for healthcare and other frontline workers. Understanding the efficacy of such methods on pathogens other than SARS-CoV-2 that may be present on PPE in healthcare settings is key to worker safety, as some pathogenic bacteria are more heat resistant than SARS-CoV-2. We assessed the efficacy of dry heat treatment against *Clostridioides difficile* spores and *Mycobacterium tuberculosis* (*M. tb*) on filtering facepiece respirator (FFR) coupons in two inoculums. Soil load (mimicking respiratory secretions) and deionized water was used for *C. difficile*, whereas, soil load and PBS and Tween mixture was used for *M. tb*. Dry heat treatment at 85 °C for 240 min resulted in a reduction equivalent to 6.0-log_10_ CFU and 7.3-log_10_ CFU in *C. difficile* spores inoculated in soil load and deionized water, respectively. Conversely, treatment at 75 °C for 240 min led to 4.6-log_10_ CFU reductions in both soil load and deionized water. *C. difficile* inactivation was higher by >1.5-log_10_ CFU in deionized water as compared to soil load (*p* < 0.0001), indicating the latter has a protective effect on bacterial spore inactivation at 85 °C. For *M. tb*, heat treatment at 75 °C for 90 min and 85 °C for 30 min led to 8-log_10_ reduction with or without soil load. Heat treatment near the estimated maximal operating temperatures of FFR materials (which would readily eliminate SARS-CoV-2) did not achieve complete inactivation of *C. difficile* spores but was successful against *M. tb*. The clinical relevance of surviving *C. difficile* spores when subjected to heat treatment remains unclear. Given this, any disinfection method of PPE for potential reuse must ensure the discarding of any PPE, potentially contaminated with *C. difficile* spores, to ensure the safety of healthcare workers.

## 1. Introduction

Personal protective equipment (PPE) is paramount to safeguard healthcare and other frontline workers from infection and transmission of SARS-CoV-2, the causative agent of COVID-19. Global supply chain disruptions and increased demand have compromised PPE availability [1], leading to considerations of methods to ensure adequate supply for frontline workers [2]. PPE may be suitable for reuse if adequately disinfected against SARS-CoV-2 and other pathogens. It has been demonstrated that SARS-CoV-2 can be successfully inactivated on coupons from filtering facepiece respirators (FFRs) using dry heat [3]. However, disinfected PPE could only be safely reused without specific restrictions if the proposed treatment protocol is also effective against other important human pathogens, in particular bacteria, which are more resistant to heat stress.

Thermal disinfection is mainly classified into two forms: moist/steam disinfection and dry heat disinfection. Dry heat disinfection is typically used in cases where the surfaces/materials subjected to treatment could be either vulnerable to damage or impenetrable to moist heat [4]. Dry heat disinfection has been also reported to be successful in microbial reduction on materials of spacecraft hardware to prevent the forward contamination of planetary bodies with living species from Earth [5]. The mechanism of inactivation of resistant bacterial spores through dry heat in a vacuum follows a different mechanism to that of moist heat inactivation, including a significant difference in survivor curves (log_10_ colony-forming units (CFU)/unit vs. time) [6]. The inactivation of bacterial spores using dry heat is mediated through cellular damage as a result of desiccation, leading to damage of biomolecules such as DNA, lipids, carbohydrates, proteins, and peptidoglycans, which compose the outer and inner layers and cortex of the spores [7]. These structural changes lead to functional changes, including altered selective membrane permeability, and alteration of genetic information, leading to cell death or mutation [7]. It is also postulated that dry heat can sub-lethally damage the inner membrane, which can lead to cell death on prolonged exposure [8]. Further, the survival of bacterial spores depends on the efficiency of DNA repair after rehydration and germination [7].

*Clostridium difficile* is a Gram-positive, anaerobic spore-forming bacillus that was officially renamed as *Clostridioides difficile* in 2016 [9]. The pathogen is widely present in the environment, though the vegetative cells need strict anaerobic conditions to proliferate [10]. Spores of *C. difficile* are tolerant to oxygen and are generally transmitted by the faecal–oral route [10]. The prevalence of *C. difficile* infection in humans increased markedly since the 18th century, especially as the most common causative agent of nosocomial infections that still affect many hospital wards, which has been partially attributed to the increasing antibiotic resistance by this spore-former in recent decades [9,11]. More than 400 different *C. difficile* strains have been reported to date, but not all are known to produce toxins and cause disease [12]. Based on clinical studies and animal models, toxigenic ribotypes of *C. difficile* have been reported so far to produce either one or two major exotoxins: toxin A (TcdA) and toxin B (TcdB) [13]. Sporulation in *C. difficile* is not very well understood but is presumed to be triggered by environmental stimuli such as nutrient limitation, quorum sensing, and other stress factors such as the presence of oxygen [14]. *C. difficile* spore ultrastructure is very similar to the spores of *Bacillus* and *Clostridium* genera, consisting of an external coat, outer membrane, intermembrane, and a germ cell wall that encloses the core that contains the nucleic acid (DNA) [15]. However, the outermost exosporium layer (covering the spore coat) of *C. difficile* spores is unique and different from other spore formers [14]. Transmission electron micrographs reveal that the exosporium layer of *C. difficile* spores is in direct contact with the spore coat due to the presence of hair-like projections [10,15]. While there have been studies reporting the recovery of *C. difficile* spores from various samples including stool [16], food (meat and pork) [17], and environmental samples [18], there is a lack of data on the potential effects of dry heat inactivation on *C. difficile* spores, as well as a selection of optimum media for recovery after heat stress. 

Tuberculosis (TB) is an infectious disease caused by the deposition of *Mycobacterium tuberculosis* (*M. tb*) onto lung alveolar surfaces via aerosol droplets, which often result in infection, immune response, and pathology [19]. While the symptomatology of TB in humans varies markedly and may affect blood, bone, and brain tissues, as well as immune and nervous systems, it is primarily a pulmonary disease [19]. Worldwide, it is one of the major causes of mortality, associated with 1.5 million deaths annually [20]. The Bacille Calmette–Guerin (BCG) attenuated vaccine prevents extra-pulmonary TB in children, but pulmonary TB must be treated using multiple long-term antibiotics, with drug-resistant TB being a particular problem [19,20,21,22]. In addition, the COVID-19 pandemic has resulted in increased TB deaths, largely due to disruptions to care and treatment [23], but also due to co-morbidity [24,25]. Its causative agent (*M. tb*) is an actinomycete that can resist desiccation due to a complex lipid-rich cell wall, containing peptidoglycan, mycolic acids, and many lipidoglycans [26]. The efficacy of chemical disinfection for the inactivation of mycobacteria is covered by standards such as the European standard, EN 14348 [27]. However, the efficacy of heat treatment against *M. tb* has been less reported. Bemer-Melchior et al. (1999) reported that treatment at 80 °C for 20 min did not achieve complete *M. tb* inactivation [28], but a study by Doig et al. [29] reported that complete inactivation of 74 cultures of *M. tb* was achieved with this time and temperature. However, to our knowledge, the efficacy of dry heat inactivation of *M. tb* on PPE has not been studied.

This study focused on exploring dry heat at 75 and 85 °C as inactivation regimens for FFR coupons inoculated with *C. difficile* spores or *M. tb*. These two bacterial pathogens were selected for specific reasons: *C. difficile* is a spore former, which is known to be resistant to extended moist heating at 71 °C [30], but to our knowledge the effects of dry heating has not been reported yet. Therefore, we selected two clinical strains and an environmental strain of *C. difficile* for our study to evaluate dry heat treatment. The selection of *M. tb* was also due to their relevance as a clinical concern and increased infection rates during the COVID-19 pandemic as well as the lack of studies reporting the efficacy of dry heat inactivation [24,25].

Soil load was used to simulate respiratory secretions [31], which may be present on PPE. The soil load may also act as a protective layer adding to the resistance of *C. difficile* spores to disinfection treatments. Deionized water, on the other hand, was included as a medium that confers no change to the inherent resistance of *C. difficile* spores. Heat treatment from 30 to 240 min at 75 and 85 °C was chosen as these temperatures were considered to be low enough to maintain FFR integrity but high enough to achieve some killing of the target bacteria. Culture media for *M. tb*, post heat treatment, used both solid selective enrichment agar and mycobacteria growth indicator tube (MGIT™) enrichment broth. Such broth was used to detect small numbers of bacteria more quickly than using solid culture alone [32]. *C. difficile* spores on the coupons were recovered on two types of plates for assessment; selective media plates for *C. difficile* and non-selective BHI plates enriched with bile salts and yeast extract. Selective media enables the proliferation of specifically targeted microorganisms and suppresses unintended microorganisms on the medium, whereas non-selective medium does not specifically restrict the growth of any microbes [33]. The selective medium is composed of a basic medium, along with specific antibiotics, chemicals, dyes, antiseptics, and sodium salts to aid in the proliferation of specific bacterial strains [33]. The use of selective and non-selective media plating for a comparative assessment of the growth of thermally treated bacteria has been widely used to understand sublethal injury, where the damage can hinder the growth of bacteria under specific conditions, but the injury is reversible in most of the cases. The incorporation of selective agents that increase osmotic pressure (such as sodium chloride or potassium chloride) in the recovery agar/media can inhibit the growth of damaged bacterial cells, that contain damaged cell organelles such as envelopes after mild thermal treatments [34]. In the current study, the enumeration/assessment of *C. difficile* spores was done on selective and non-selective media to compare their growth and detect sub-lethally injured cells (if any). 

## 2. Materials and Methods

### 2.1. Preparation of Inoculums

#### 2.1.1. Preparation of *C. difficile* Spore Suspension 

Two clinical strains (*C. difficile* NZRM3605 and *C. difficile* NZRM2377) and an environmental strain (*C. difficile* NZRM2390) were obtained from the New Zealand Reference Culture Collection: Medical Section (NZRM) (Wellington, New Zealand) and revived in 10 mL of tryptic soy broth (TSB) (Fort Richard Laboratories, Auckland, New Zealand). Two clinical and one environmental strain was selected to cover a broader range for source of origin for this pathogen based on the recent publications that indicate that *C. difficile* can be either nosocomial or a community contaminant from the environment [18,35]. The inoculated TSB cultures were incubated for 24 h at 37 °C for 48 h inside an anaerobic chamber (Don Whitley Scientific, Yorkshire, UK) (gas phase) with a gas composition of CO_2_ (10%), H_2_ (5%), and N_2_ (85%) with a relative humidity set at 70%. The bacterial culture was then streaked onto pre-reduced Columbia sheep blood agar (SBA) and tryptic soy agar (TSA) (both Fort Richard Laboratories) plates, followed by further incubation for 48 h in similar anaerobic conditions at 37 °C. Spore culture and harvest were conducted according to the nutrient exhaustion method reported by Rodriguez-Palacios et al. [30]. A loopful of colonies from each strain were individually inoculated in TSB and further incubated for 48 h in anaerobic conditions at 37 °C. Inoculated cultures were then spread-plated (200 μL) onto pre-reduced Brain Heart Infusion (BHI) plates (Fort Richard Laboratories) supplemented with 5% (*w*/*v*) yeast extract (Fort Richard Laboratories) and 0.1% (*w*/*v*) of bile salts (Sigma Aldrich, Auckland, New Zealand). The plates were prepared as described previously [36], with a slight modification. As sodium taurocholate, an essential bile salt required to induce germination of *C. difficile* spores [37], was not being supplied to New Zealand at the time of the study during the COVID-19 pandemic, it was replaced with bile salts (Sigma-Aldrich). The inoculated plates were incubated in an anaerobic chamber for 6 days at 37 °C, followed by transfer to an incubator set at 25 °C in aerobic conditions while secured in zipper bags and incubated for an additional 6 days. This step aimed to induce stress due to the presence of oxygen, lack of nutrients, as well as temperature stress (below the bacteria’s optimum range). After 6 days, the colonies on these plates were scraped off using a sterile L-shaped spreader by adding 1 mL of sterile Milli Q water. The spores were harvested by centrifugation (7000× *g* for 10 min) at 4 °C, resuspending in chilled Milli Q water followed by washing two more times (7000× *g* for 4 min). The three different strains were maintained and washed separately throughout the procedure and were separately enumerated on pre-reduced enriched BHI agar plates (supplemented with 5% (*w*/*v*) yeast extract and 0.1% (*w*/*v*) of bile salts) and CHROMID^®^ *C. difficile* agar plates (Fort Richard Laboratories) as described. CHROMID^®^ *C. difficile* agar consists of a rich nutritive base combining different peptones and taurocholate, which favours the germination of spores, and a chromogenic substrate [38]. Every time before enumeration, the spores were heat-activated at 60 °C for 10 min as a step reported to be essential for inducing their germination [39]. *C. difficile* spore stock solutions were prepared in deionized sterile water (10^9^ CFU/mL), and the working solution or final inoculum was re-constituted in either deionized water or soil load as described in Section 2.2.

#### 2.1.2. Preparation of *M. tb* Solution and Media Used in the Experiment

*M. tb* H37Rv strain TMC102 was sourced originally from the Trudeau Institute (Saranac Lake, New York, NY, USA), and subsequently stored as seed cultures at −80 °C. A seed culture vial was rapidly thawed (~1 min), and 100 µL was inoculated into individual 4 × 5 mL Dubos Tween Albumin Broth (TAB; Becton Dickinson, Sparks, MD, USA) and incubated aerobically, without shaking, at 37 °C for 7 days. On day 5, the TAB cultures were checked for contamination from rapidly growing bacteria by streaking a 10-µL loopful of bacterial culture onto horse blood agar plates (Fort Richard Laboratories), which were incubated aerobically for 18 h at 37 °C. On day 7, the TAB was centrifuged at 3000× *g* for 10 min. The supernatant was discarded, and the pellet was resuspended in 0.01 M phosphate buffer saline (PBS, prepared in-house) plus 0.05% Tween 80 (Sigma Aldrich). The PBS and Tween mixture is referred to as PBST. The pellet was pre-determined to obtain 1 × 10^9^ colony-forming units (CFU) in a 5-μL inoculum. Tween-80 was used to reduce the clumping of mycobacteria.

### 2.2. Inoculation of FFR Coupons

Circular coupons (8 mm in diameter) were cut from FFRs of the same model (Help-It FFP2 respirators, QSi, Whanganui, New Zealand) as previously reported [3]. Using the pre-enumerated stock solution, a cocktail of the three strains (*C. difficile* NZRM3605 and *C. difficile* NZRM2377 and *C. difficile* NZRM2390) was prepared by combining them in equal volumes, which was further enumerated as described above, and two types of inoculums were constituted. 

#### 2.2.1. *C. difficile* Inoculation

*C. difficile* spore cocktail was inoculated in two sets: in autoclaved deionized water and soil load. The soil load simulates the presence of respiratory secretions, being composed of three protein types (high-molecular-weight proteins, low-molecular-weight peptides, and mucous material), specifically tryptone, bovine serum albumin (BSA), and porcine mucin (Sigma-Aldrich) [3]. The soil load was prepared as per the American Society for Testing and Materials (ASTM) E2197-11 standards [40].

Five 24-well sterile microtiter plates (Micro Analytix, Auckland, New Zealand) were prelabelled as PC (positive control), 30, 60, 120, and 240 min for the heat experiment against *C. difficile* at 85 °C. Each plate consisted of three types of inoculums, with 6 wells per type of inoculum: *C. difficile* in deionized water, *C. difficile* in soil load, and NC (negative controls). Using sterilised tweezers, one coupon was placed (with the side with dark-coloured thread facing upwards) in each of the 18 wells. A plate in a similar protocol as explained above was used as the PC where the incubation time was 10 min at room temperature (18–22 °C). For each trial, 12 coupons were inoculated with 5 μL of the spore suspension (as a single drop with 10^9^ CFU) that was carefully placed in the centre of each coupon (6 with *C. difficile* in deionized water and 6 in soil load), with additional 6 uninoculated coupons used as the negative control (NC). The total time taken for inoculation of 12 coupons was approximately 2 min. 

The microtiter plate was then covered with its original lid, placed inside the anaerobic box, and a timer for 10 min was started. The coupons were allowed to stand for 10 min before being exposed to dry heat. In the case of the positive control (no heat treatment), the bacterial spores were recovered and assessed as described in 2.3 after 10 min of exposure at room temperature (18–22 °C). For treatment at 85 °C, the plates in the anaerobic box with anaerobic environment generator sachets (Oxoid AnaeroGen™, Thermo Scientific, Auckland, New Zealand) were placed inside the oven pre-heated to 85 °C. At each sampling point (30, 60, 120, and 240 min), a microtiter plate was removed from the oven and allowed to cool down for 2 min at room temperature, before recovery and assessment as described in Section 2.3.1. 

#### 2.2.2. *M. tb* Inoculation

Two different inoculums were prepared: bacteria in PBST only and soil load as per ASTM E2197-11 standards [40].

Seven 24-well sterile tissue-culture plates (BD Falcon, NJ, USA) were pre-labelled to indicate temperature and heat treatment time: PC (room temperature) for 10 min, 75 °C for 30 min, 75 °C for 60 min, 75 °C for 90 min, 85 °C for 30 min, 85 °C for 60 min, 85 °C for 90 min, allowing 6 replicate wells for each of 3 treatments: *M. tb* in PBST, *M. tb* in soil load + PBST, and NC. Using sterilised tweezers, one circular coupon with the side with dark-coloured thread upwards was placed into each well of the 24-well plates. For each temperature and duration plate, coupons were inoculated with 5 μL of a solution containing 1 × 10^9^ CFU of the relevant culture (6 coupons with *M. tb* in PBS and 6 coupons with *M. tb* in soil load). Also included were 6 untainted NC coupons. 

For each of the temperature durations, a single 24-well plate was selected. It took approximately 2 min to inoculate all coupons on each plate. The PC plate was covered with its lid and allowed to stand for 10 min at room temperature to ensure absorption of the inoculum, with its bacteria harvested immediately after. Following the 10-min stand-down period, the plates to be heat-treated were placed one at a time inside the oven and exposed to dry heat treatment at 75 °C or 85 °C and the pre-established duration. Afterwards, the plate was removed from the oven and allowed to cool down for 2 min at room temperature, and the bacteria were harvested.

### 2.3. Recovery and Visual Assessment of Heat Inactivation Experiment

#### 2.3.1. Recovery of *C. difficile* Spores

Both selective and non-selective media were used for the initial assessments. Non-selective media plates were prepared as described above (Section 2.1.1). To specifically eliminate any background contaminants on the FFR coupons, the CHROMID^®^ *C. difficile* Agar plates (Fort Richard Laboratories) were used, which are the chromogenic media for the detection and identification of *C. difficile*. These *C. difficile* selective media plates consist of a rich nutritive base combining different peptones and taurocholate that favors the germination of its spores. At each sampling point, a cooling time of 2 min was included followed by the transfer of each coupon using sterile forceps to a pre-labelled 2-mL Eppendorf tube containing 500 μL of PBS. The coupon suspension was vortexed for 30 s, then transferred into an anaerobic chamber where 4 × 50 μL of the suspension were spotted onto a pre-labelled pre-reduced BHI plate, with a further 4 × 50 μL spotted onto the pre-reduced selective media plate for *C. difficile* spores. The coupon (soaked with the remaining PBS) was also placed on the selective media plate, and the inoculated plates were left in the same position for 24 h at 37 °C in the anaerobic chamber. These were subsequently moved into anaerobic boxes with anaerobic environment generator packs (Oxoid AnaeroGen™), where they were incubated for an additional 48 h to determine bacterial survival. Survival plates were also imaged. 

#### 2.3.2. Recovery of *M. tb*

Following heat treatment, each coupon was transferred to a sterile microcentrifuge tube containing 1000 μL PBS and vortexed for 30 s. Four × 100 µL of this PBS solution were then spotted onto a single Middlebrook 7H11 agar plate (Becton Dickinson, Sparks, MD, USA). This media had been supplemented with glycerol and Oleic Albumin Dextrose Catalase (OADC; Sigma, St. Louis, MO, USA) enrichment. In addition, 500 µL was transferred to a Mycobacteria Growth Indicator Tube (MGIT; Becton Dickinson), containing BBL^TM^ MGIT broth in a cocktail of OADC enrichment and polymyxin-B, amphotericin-B, nalidixic acid, trimethoprim, and azilocillin (PANTA) antibiotic (Becton Dickinson). 

All media were incubated at 37 °C for 3 weeks to determine bacterial survival. Growth was confirmed as mycobacteria based on colony morphology and acid fastness after Ziehl–Neelsen staining (using reagents prepared in-house). In addition, growth detected in the MGIT tube was confirmed using the criterium of acid-fast staining, but no growth was detected on horse blood agar after 2 days of incubation. Survival plates were imaged.

### 2.4. Quantification of Reduction in *C. difficile* Spores by Dry Heat Treatment at 75 and 85 °C

The spores were harvested after heat treatment at either 75 or 85 °C for 90, 180, or 240 min, as described in Section 2.3.1. Sterile forceps were used to transfer each coupon to a pre-labelled Eppendorf tube (2 mL) containing 1000 μL of PBS, vortexed for 30 s, followed by ten-fold serial dilutions up to 1:100,000, followed by drop plating (10 μL) onto *C. difficile* CHROMID^®^ selective media plates (Fort Richard Laboratories). The plates were then incubated in an inverted position at 37 °C for 3 days in an anaerobic box, with anaerobic environment generator sachets (Oxoid AnaeroGen™) and an indicator strip (BBL™ GasPak™ Anaerobic Indicator Strip, Dry, Fort Richard Laboratories) before enumeration.

### 2.5. Temperature Monitoring 

The temperature inside the oven was constantly monitored and recorded using a Lascar digital data logger (EL-USB-2-LCD, Lascar Electronics, Hong Kong, China) and Tinytags dataloggers (Gemini Data Loggers Ltd., Chichester, UK). The Lascar data logger was set to the maximum logging rate of one measurement every 10 s. For the heat treatment, a Forced-Air Type oven (Daihan, Interlab Ltd., Porirua, New Zealand) was used, which was pre-calibrated and pre-set at the desired temperature (75 or 85 °C) before a given treatment plate was deposited inside it. The graphs showing the temperature profiles inside the oven are provided in the Supplementary Material (Figures S1–S4). Variations of not more than ±1 °C around the target temperature were observed throughout all heat treatment cycles.

### 2.6. Statistical Analysis

The magnitude of the reduction in *C. difficile* spore numbers as a result of dry heat treatment was compared using a linear mixed model. It included temperature (75 and 85 °C), inoculum type (soil load and deionized water), treatment duration (90, 180, and 240 min), and a 3-way interaction term as predictors, with the set of three replicates, also included as a random factor. The interaction term was statistically significant at *p* < 0.0001 and thus retained in the model, with the resulting least-squares means (adjusted means) and respective 99% confidence intervals reported as the outcomes of interest. The data analysis was performed in SAS v9.4 (SAS Institute, Cary, NC, USA) with α set at 1% as the threshold for statistical significance. The associated results were graphically illustrated with Microsoft Excel. 

## 3. Results

### 3.1. Visual Assessment of Inactivation of *C. difficile* at 85 °C

As assessed on the selective media plates, exposure up to 240 min at 85 °C did not lead to the complete elimination of *C. difficile* spores across all replicates in both soil load and deionized water (Table 1). However, the non-selective media plates were not capable of recovering the heat-treated spores at several time points (Table 1). 

**Figure 1:**
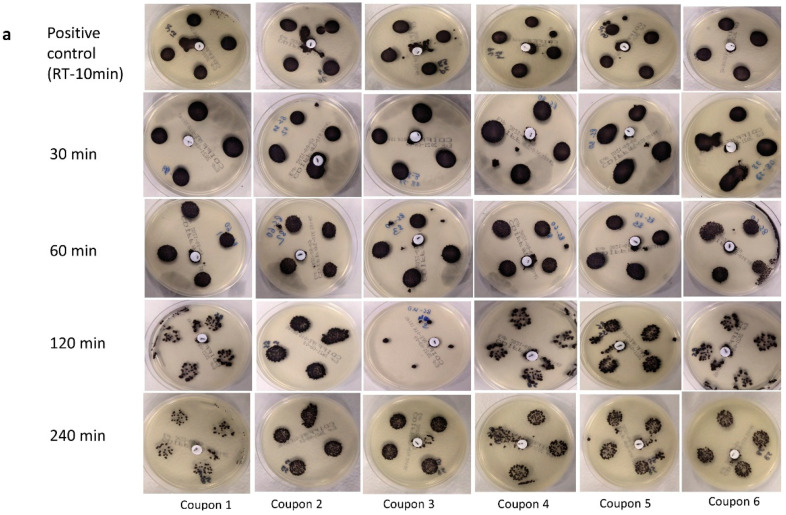

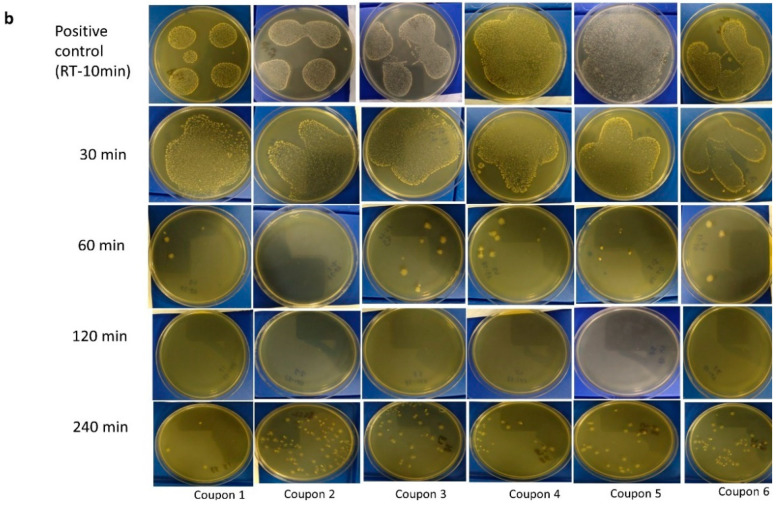
Presence/absence of *C. difficile* spores recovered on selective media (**a**) and non-selective media (**b**) on the positive controls (room temperature at 18–22 °C for 10 min) and after dry heat treatment at 85 °C for 30, 60, 120, and 240 min, while being suspended in soil load and inoculated onto filtering facepiece respirator (FFR) coupons. Each test was carried out on 6 coupons to ensure replication.

The results obtained on non-selective media plates after 120 min of dry heat treatment in both deionized water and soil load were unexpected, as the recovery was very low or none even though conditions of treatment and recovery were identical across all treatment durations. Hence, the reasons behind these findings are unclear. Nonetheless, some spores were recovered after 240 min of treatment in both deionized water and soil load on non-selective media plates, indicating that the treatment duration was not sufficient to render complete inactivation (9-log_10_ reduction) on any coupons. The inconsistency in recovery on non-selective media plates indicates that only selective media plates could recover the spores post-treatment with acceptable reproducibility. For each treatment protocol a positive control was included (incubation at room temperature for 10 min), and colony densities on both selective and non-selective media were largely similar (Figure 1a,b). When inoculated in soil load, treatment at 85 °C for 30 min led to no apparent reduction in the density on either selective (Figure 1a) or non-selective plates (Figure 1b). The colonies on CHROMID^®^ *C. difficile* Agar were grey-black as expected for *C. difficile* [38]. The density of colonies on selective media was visibly higher than that on non-selective media plates. 

**Figure 2:**
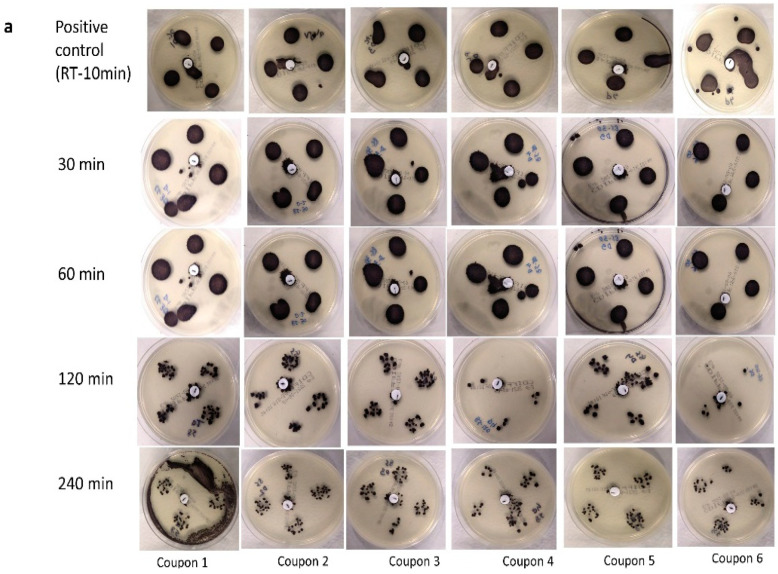

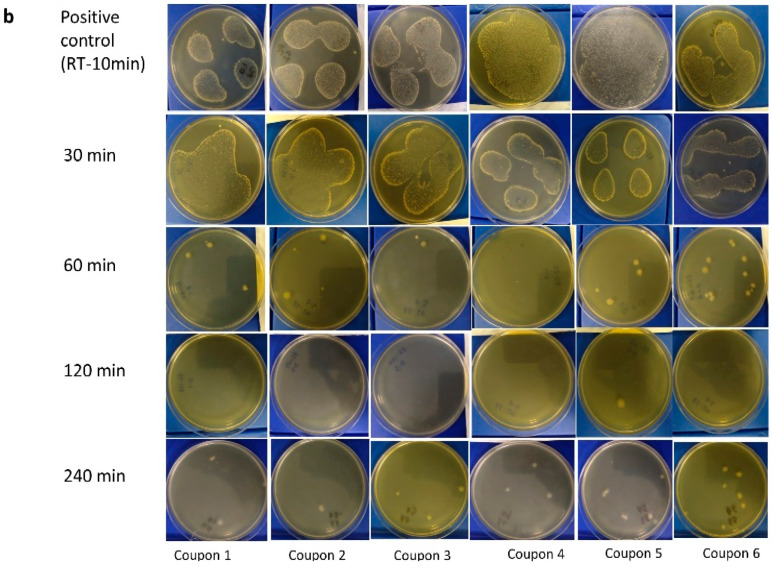
Presence/absence of *C. difficile* spores recovered on selective media (**a**) and non-selective media (**b**) on the positive controls (room temperature at 18–22 °C for 10 min) and after dry heat treatment at 30, 60, 120 and 240 min, while being suspended in deionized water and inoculated onto filtering facepiece respirator (FFR) coupons. Each test was carried out on 6 coupons to ensure replication.

The experiments using deionized water as a medium for inoculation yielded similar results, where treatment up to 240 min could not eliminate the spores (Figure 2a,b). However, *C. difficile* colonies were less dense than in soil load (based on visual assessments), especially after 120 and 240 min. Again, using non-selective media, the spores in deionized water subjected to heat treatment at 85 °C for 120 min were not recovered on 5/6 plates (Figure 2b). However, contrasting results were obtained after 240 min of treatment, where at least one colony was recovered on the non-selective media and was therefore designated as positive (Figure 2b). 

These experiments yielded two key findings:

1. Dry heat treatment at 85 °C for up to 240 min did not result in complete elimination of the *C. difficile* spores, although the increase in temperature did indicate some reduction in the density of colonies upon visual inspection, with the presence of soil load having a protective effect.
2. Non-selective media were unreliable for the recovery of heat-treated *C. difficile* spores. Hence, only selective media were used for the subsequent experiments in this study.

### 3.2. Quantitative Estimation of Inactivation of *C. difficile* Spores 

**Figure 3:**
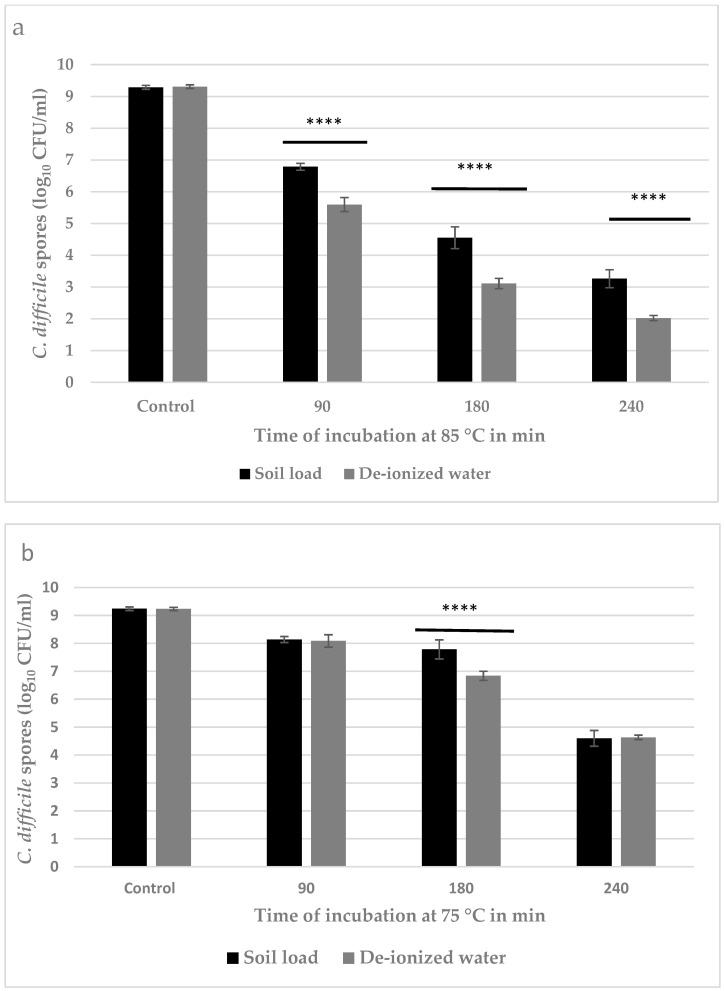
Inactivation of *C. difficile* spore numbers using dry heat treatment at 85 °C (**a**) and 75 °C (**b**) in a soil load (black bars) and deionized water (grey bars) inoculums. Data are the least-squares means (adjusted means) with error bars representing the respective 99% confidence intervals. **** *p* < 0.0001 for a difference between inoculum types at a given treatment duration. At each heat treatment temperature, all pairwise comparisons between treatment durations for a given inoculum type were statistically significant at *p* < 0.0001. Further, for the given treatment duration and inoculum type, all differences in the magnitude of inactivation between 75 and 85 °C were statistically significant at *p* < 0.0001. Controls indicate “no thermal treatment”.

Based on the visual assessment, complete inactivation of *C. difficile* spores was not achieved by dry heat treatment at 85 °C. However, there was a visible reduction in the density of surviving spores after 120 and 240 min of heat treatment compared to 30 and 60 min and positive controls. Therefore, separate trials were conducted to quantify the reduction in *C. difficile* spores achieved by dry heat treatment at 75 and 85 °C for 90, 180, and 240 min. Heat treatment at 85 °C for 240 min led to a reduction in *C. difficile* spores by log_10_ 7.3 ± 0.1 CFU/coupon and log_10_ 6.0 ± 0.1 CFU/coupon in deionized water and soil load, respectively (Figure 3a). The reduction in the number of spores after dry heat treatment at 75 °C was log_10_ 4.6 ± 0.1 in both deionized water and soil load (Figure 3b). 

It was observed that when *C. difficile* spores were heat treated at 85 °C, the number of viable spores progressively decreased with increasing duration of treatment in both deionized water and soil load (Figure 3a). Additionally, at 85 °C, the number of surviving *C. difficile* spores was markedly lower (0.5–1.0 Log_10_ CFU/coupon) in deionized water compared to soil load irrespective of treatment duration (Figure 3a). However, with dry heat treatment at 75 °C, there were no differences in inactivation levels of *C. difficile* spores between the deionized water and soil load after 90 min and 240 min (Figure 3b). However, after 180 min at 75 °C, there was greater inactivation (+1 Log_10_ CFU/coupon; *p* < 0.05) in deionized water (Figure 3b). 

### 3.3. Visual Assessment of M. tb Inactivation at Room Temperature, 75 °C, and 85 °C on Agar

The results in Table 2 show that bacteria in the positive controls grew as expected. Heat treatment at 75 °C showed there was *M. tb* growth in 4/6 and 2/6 of the coupons inoculated with bacteria only after 30 and 60 min of treatment, respectively (Table 2). No *M. tb* growth was seen in any of the heat-treated FFR coupons inoculated with the bacteria in soil load, irrespective of treatment duration (Table 2). On agar, dry heat treatment at 85 °C led to complete inactivation of *M. tb* on FFR coupons (Table 2) with no growth of bacteria at any of the time points. All plates used for the visual assessment of *M. tb* growth during the experiments are shown in Table S1 in the Supplementary Material. A confluent lawn of growth was seen from each of the 4 × 100µL aliquots placed on all plates with positive controls (Table S1 in Supplementary Material). Two plates had excessive moisture resulting in a smear of growth rather than discrete drops as usually observed (Table S1 in Supplementary Material). Although this experiment aimed to determine the presence or absence of growth, it was still possible to quantify *M. tb* reduction in those instances where growth was observed because each coupon was inoculated with 10^9^ bacteria, and after heat treatment a maximum of 10^1^ bacteria were seen on culture, being a minimum 8-log_10_ reduction.

### 3.4. MGIT Enrichment Broth

Little *M. tb* growth was visually detectable on agar with the naked eye (Table 2). However, the subsequent use of MGIT enrichment broth to resuscitate bacteria yielded different results with *M. tb* growth observed in at least 4/6 replicates after dry heat treatment at 75 °C for 30 or 60 min (Table 3). While markedly higher rates of *M. tb* inactivation were achieved after 90 min at 75 °C, there was no complete inactivation, with *M. tb* growth observed in at least 1/6 coupons (Table 3). At 85 °C, heat treatment for 30 or 60 min was still insufficient to eliminate *M. tb*, but treatment for 90 min led to complete inactivation (Table 3). Of note, there was no observed effect of inoculum type (soil) on the efficacy of heat treatment against *M. tb*.

## 4. Discussion

### 4.1. Inactivation of C. difficile Spores

Thermal resistance in bacterial spores can be classified as resistance towards either wet or dry heat treatment. Wet heat resistance is mainly attributed to the water content and mineral ions in the core, stability of spore proteins, and saturation of alpha/beta-type small, acid-soluble spore proteins (SASP) that protect DNA against wet heat damage [41]. The resistance to dry heat on the other hand has been less commonly reported and is not well understood. However, the role of the alpha/beta-type SASP in spore resistance to dry heat is well established as the major mechanism (reported so far) involved in DNA repair [7,41]. This further supports that dry heat leads to specific damage by increasing the mutational frequency of the spore DNA, and hence loss of viability [42].

Although the potential mechanisms behind dry heat resistance have not been investigated specifically in *C. difficile* spores, certain DNA repair mechanisms such as RecA-dependent DNA repair systems, *exoA*, *endoIV* (*yqfS*) and the products of the ykoUVW operon, and *σG* for RNA polymerase have been well-described in *Bacillus subtilis* spores [43,44]. The role of *dnaK* as a response to moist heat treatment has been reported in *C. difficile* [45]. Although the mechanism of moist and dry heat response might not be similar, these reports indicate potential pathways that could be investigated. In *C. difficile* spores, the *dnaK* gene leads to the translational product Chaperone protein DnaK, which is known to be expressed in heat-induced stress, and the *dnaK* mutant *C. difficile* strain 630 has been reported to exhibit temperature sensitivity, slower growth, and reduced thermotolerance [45,46]. The function of the DnaK chaperone that has been reported in *Escherichia coli* is to remodel the DNA polymerase/helicase complex to permit repair [46]. A study by Jain et al. reported the first comparative proteomic analysis of the heat stress (41 °C) response in vegetative cells of *C. difficile* strain 630, to that of existing proteomics data sets, and indicated that classical heat shock proteins including GroEL, GroES, DnaK, Clp proteases, and HtpG were up-regulated [45]. Up-regulation of hydrogenases and various oxidoreductases suggests that electron flux across these pools of enzymes changes under heat stress. However, any potential association with the thermal resistance exhibited by spores of this strain was not reported [45]. 

Results obtained in the current study indicated the inability of the heat-treated/stressed spores to be recovered/quantified on non-selective media plates that were formulated as described previously [36], with a slight modification that was the replacement of sodium taurocholate by bile salts. This did not effectively reduce the recovery of untreated/dormant spores; however, once heat treated, especially above 60 min at 85 °C, the spores did not germinate and colonize on these plates, whereas the selective media plates with sodium taurocholate were capable of recovering these spores. These findings support the previously reported role of sodium taurocholate in the repair of heat-stressed spores, where it prevents the spore coat from being a barrier for the non-nutrient-based germinants [47]. Taurocholate is one of the conjugated bile acids that is secreted in the gastrointestinal (GI) tract (from the liver) and has been reported to interact with *C. difficile* spores in the tract as an inducer to initiate germination [48]. Heat treatment at 80 °C for 10 min has reportedly reduced the recovery rates of *C. difficile* spores on BHI agar medium by 95–99%, but the recovery was enhanced by the addition of sodium taurocholate [49]. Therefore, for the efficient recovery of *C. difficile* spores post-heat treatment, it is recommended to use more than one type of media to ensure the detection of all the surviving viable spores. Additionally, the presence of sodium taurocholate in the recovery media is essential to induce germination in stressed spores and for the repair of spores after sublethal injury before colonization. CHROMID^®^ *C. difficile* Agar consists of a rich nutritive base combining different peptones and taurocholate, which favours the germination of spores, and a chromogenic substrate [38], and a mixture of antibiotics that enables the detection and identification of β-glucosidase-producing *C. difficile* strains, identified by the typical grey to black colour of colonies [50]. The inconsistency in the recovery of colonies on the non-selective media reemphasises the requirement of the specific selective medium for detection of *C. difficile* spores, especially after sublethal heat treatments.

Our experiments showed that dry heat treatment at 85 °C for ≥120 min would reduce numbers of *C. difficile* spores on PPE up by at least 6-log_10_ CFU/coupon, while a CFU reduction of ~4.5-log_10_ was achieved after 240 min at 75 °C. The unavailability of data in the literature makes it difficult to directly compare and validate our findings. Decimal reduction time (D-value) can be defined as the time required at a temperature to reduce the spore population by 90%. D-values of *C. difficile* spores in peptone water (0.1%) were found to be 1.42 to 3.82 min at 85 °C and 4.9 to 5.9 h at 75 °C [47]. The marked differences reported [47] in D-values (and hence inactivation efficacy) between the two temperatures only 10 °C apart were not observed in our current study, suggesting that resistance to dry and wet heat is unlikely to be comparable for any bacterial spore strain. 

### 4.2. Inactivation of M. tb

*M. tb* does not sporulate, so results were limited to the growth of vegetative microorganisms. The presence of any viable bacteria would likely pose a health risk of tuberculosis infection. *M. tb* is slow-growing, so the use of selective and enrichment media is important to determine the presence of viable bacteria persisting in small numbers. In this study, there was a minimum 8-log_10_ reduction of *M. tb* when tested on agar, after 30 min at 75 °C. but some viable *M. tb* remained, which could result in an unacceptable level of risk for those wearing heat-treated PPE that was heavily contaminated with this bacterium beforehand. Increasing the time of treatment at 75 °C for 90 min inactivated *M. tb* using agar to detect growth. If dry heat treatment at 85 °C was used, an 8-log_10_ reduction was achieved at all assessed treatment durations (≥30 min). The higher temperature of 85 °C for 90 min ensured no growth on agar or in enrichment broth. This would be a preferable (maximum) temperature to use to minimize the likelihood of adverse effects on the integrity and function of treated PPE, which could otherwise compromise the safety of the wearer Surprisingly, the presence of soil load did not appear to protect *M. tb*, as no growth was observed at either 75 or 85 °C. While the protein content of the soil load is generally thought to be protective, a study by Belcher et al. showed that some mycobacterial strains were unable to bind mucin [51], binding directly with the mucosal epithelium in vivo. In our vitro experiment, it is likely that the presence of mucin provided a moist heat environment, allowing for faster rapid heat exchange and, consequently, more rapid killing. In a clinical setting, there would likely be a time delay (of hours, or possibly days) between PPE contamination and heat treatment. During this time, there would be some level of *M. tb* inactivation due to environmental factors. However, this study did not investigate the effect of time between PPE inoculation and heat treatment on bacterial survival [52]. The infectivity and transmission of *M. tb* are complex, but risks can be minimized [53]. While the growth of *M. tb* in enrichment MGIT broth indicates a theoretical infection risk, it would be very dependent on the initial load of bacteria on PPE. PPE with any sign of visual contamination or that had been used in contact with known or suspected TB cases should be directly disposed of and not sent for recycling and reuse. Otherwise, the use of heat-treated PPE would most likely pose a potential risk of infection to the wearer.

## 5. Conclusions

Inactivation of *C. difficile* spores was directly influenced by the duration of exposure at 75 and 85 °C, with greater levels of inactivation achieved at the higher temperature. Soil load had a protective effect on the *C. difficile* spores leading to reduced inactivation in most cases. A reduction equivalent to 6-log_10_ CFU/coupon in *C. difficile* spores was achieved at 85 °C after 240 min, and this reduction was enhanced in the absence of soil load in the matrix. This indicates the possibility of using an accurate time–temperature combination of dry heat for disinfection of PPE against *C. difficile* spores. However, the extended exposure times could damage the structural elements and thus the integrity of PPE and hence might need to be addressed separately. The use of specific selective media can be deemed essential for the accurate detection and recovery of *C. difficile* spores. Future extension of this work will investigate the mechanism of dry heat resistance depicted by *C. difficile* spores with a close comparison to the genes and pathways involved in moist heat resistance. There was a minimum 8-log_10_ reduction of *M. tb* on agar with heat treatment for 30 min at 75 °C. However, the growth of *M. tb* in MGIT liquid media post-treatment at 75 °C indicated that small numbers of bacteria survived after heat treatment. While there were fewer positive cultures after 30 and 60 min at 85 °C regardless of the presence of the soil load, complete killing was not observed without at least 90 min of heat treatment at 85 °C. Therefore, this would be the safest temperature for the heat treatment of PPE, possibly contaminated with *M. tb*. Material integrity of PPE post-heat disinfection is the subject of future research.

## Figures and Tables

**Table 1:** Presence/absence of *C. difficile* spores based on visual inspection of inoculated coupons in soil load and deionized water after dry heat treatment at 85 °C for 30, 60, 120, and 240 min.

Temperature	Time (min)	Type of Media	Inoculum Type		
			Soil Load	Deionized Water	NC
PC	10	Non-selective	6/6	6/6	0/6
		Selective	6/6	6/6	0/6
85 °C	30	Non-selective	6/6	6/6	0/6
		Selective	6/6	6/6	0/6
	60	Non-selective	5/6	5/6	0/6
		Selective	6/6	6/6	0/6
	120	Non-selective	0/6	1/6	0/6
		Selective	6/6	6/6	0/6
	240	Non-selective	6/6	6/6	0/6
		Selective	6/6	6/6	0/6

Note: Each test was carried out on 6 coupons to ensure replication. Positive controls (PC) were left to stand for 10 min at room temperature (18–22 °C). NC, negative controls.

**Table 2:** Growth of *Mycobacterium tuberculosis* (*M. tb.*) on 7H11G agar after different dry heat treatment regimens, and subsequent incubation for 3 weeks at 37 °C.

Temperature	Time (min)	Inoculum Type		
		*M. tb.* Only	Soil Load	NC
PC	10	6/6	6/6	0/6
75 °C	30	4/6	0/6	0/6
	60	2/6	0/6	0/6
	90	0/6	0/6	0/6
85 °C	30	0/5 ^†^	0/6	0/6
	60	0/6	0/5 ^†^	0/6
	90	0/6	0/6	0/6

NC, untainted negative controls; PC, positive controls kept at room temperature for 10 min (≈18–22 °C). Data are the number of plates with observed *M. tb* growth out of 6 replicates. ^†^ Data for these two sets of replicates are reported out of 5 rather than 6 coupons, due to cross-contamination with rapidly growing non-mycobacterial bacteria.

**Table 3:** Growth of *Mycobacterium tuberculosis* (*M. tb*) in MGIT enrichment broth after different dry heat treatment regimens, and subsequent incubation for 3 weeks at 37 °C.

Temperature	Duration (min)	Inoculum Type		
		*M. tb.* Alone	Soil Load	NC
PC	10	6/6	6/6	0/6
75 °C	30	5/6	4/6	0/6
	60	4/6	5/6	0/6
	90	1/6	1/6	0/6
85 °C	30	3/6	5/6	0/6
	60	2/6	3/6	0/6
	90	0/6	0/6	0/6

NC, untainted negative controls; PC, positive controls kept at room temperature for 10 min (18–22 °C). Data are the number of tubes with observed *M. tb* growth out of 6 replicates.

## Data Availability

Not applicable.

## Supplementary Materials

The following supporting information can be downloaded at: https://www.mdpi.com/article/10.3390/pathogens11080871/s1, Figure S1: Temperature profile for inactivation at 85 °C for 240 mins using Lascar data Logger; Figure S2: Temperature profile for inactivation at 85 °C up to 120 mins using Lascar data logger, Figure S3: Temperature profile for the log-reduction assay at 75 °C for up to 240 min measured with a Tiny Tag Logger; Figure S4: Temperature profile for the log-reduction assay at 85 °C for up to 240 min measured with a Tiny Tag Logger; Table S1: Visual assessment of *Mycobacterium tuberculosis* (*M. tb*.) on 7HllP agar plate after different dry heat treatment regimens, and subsequent incubation for 3 weeks at 37 °C.
