# Supplementary material for: Efficacy of Dry Heat Treatment against Clostridioides difficile Spores and Mycobacterium tuberculosis on Filtering Facepiece Respirators
Source: Pathogens. 2022 Aug 2;11(8):871. doi: 10.3390/pathogens11080871 (PMC9415841; doi:10.3390/pathogens11080871)
Supplement: Supplementary file 1 [file pathogens-11-00871-s001.zip › pathogens-1820956-supplementary.pdf]

pathogens

MDPI

# Supplementary Material

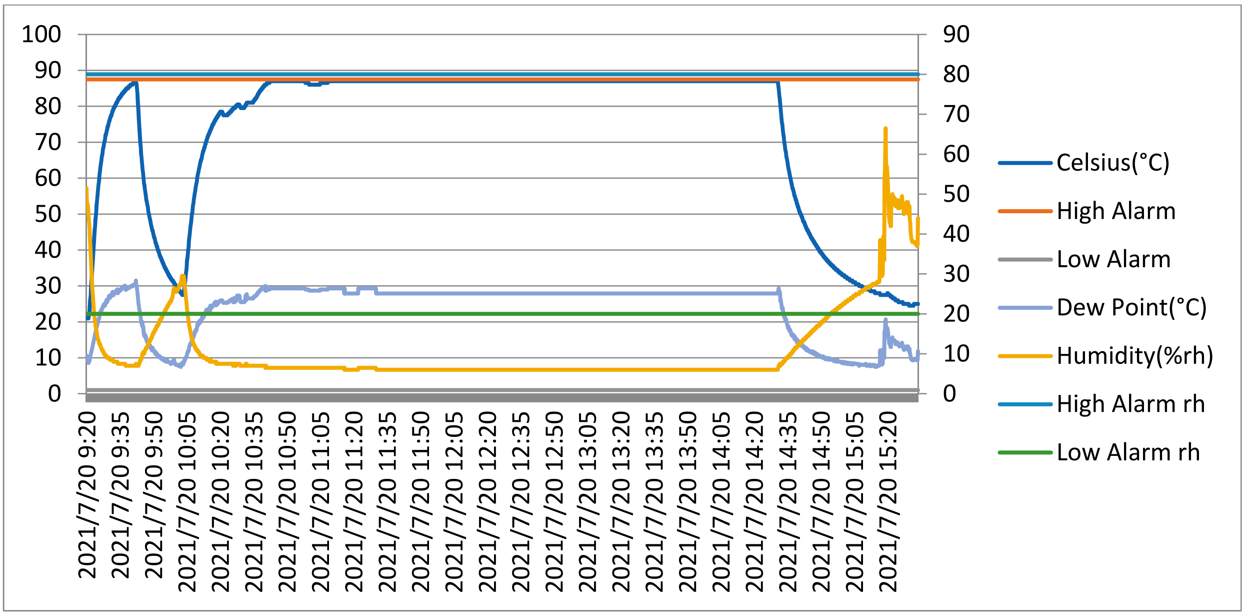


**Figure S1**: Temperature profile for inactivation at 85 °C for 240 min recorded using a Lascar data logger.High alarm was set at 97 ºC and Low alarm was set as 23 ºC.

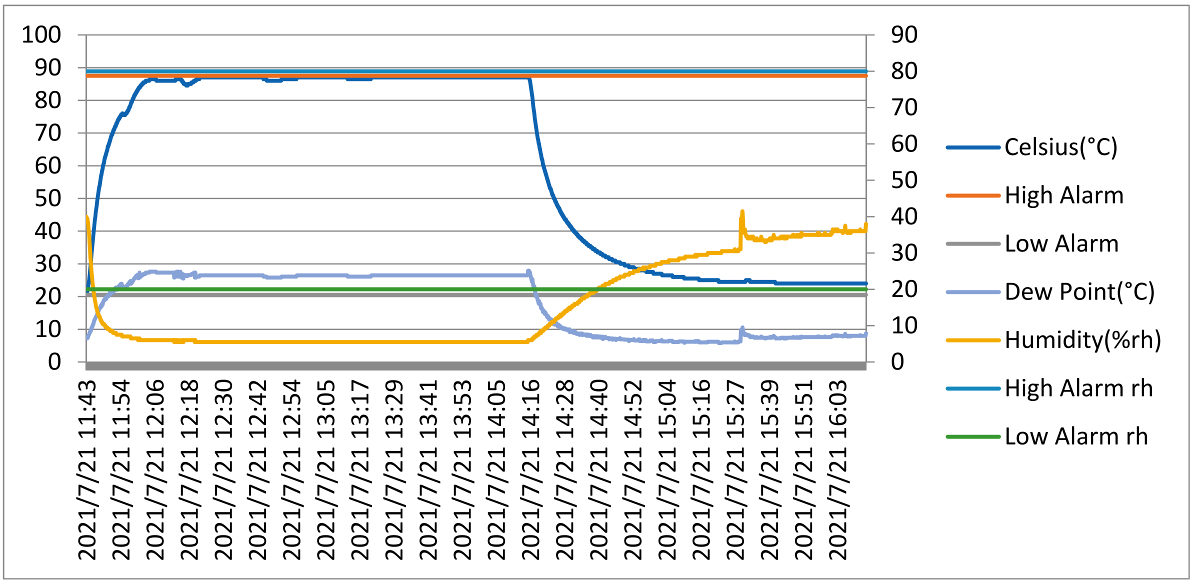


**Figure** S**2:** Temperature profile for inactivation at 85 °C up to 120 min using a Lascar data logger.

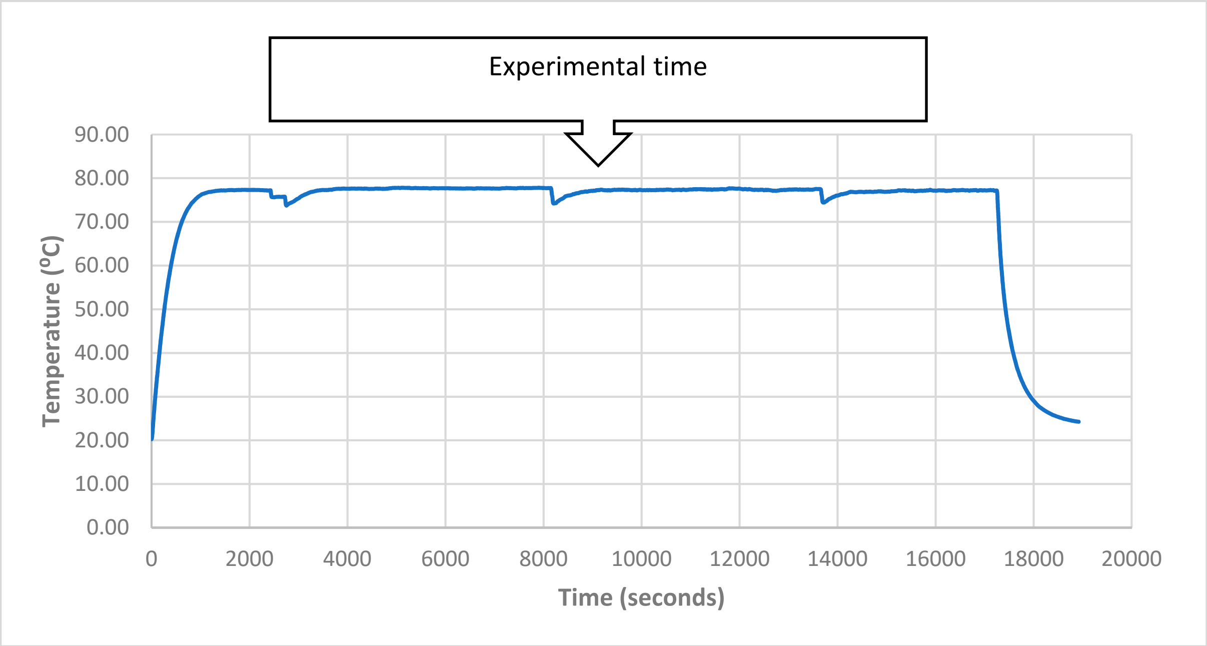


**Figure S3**: Temperature profile for the log-reduction assay at 75 °C for up to 240 min measured with a Tinytag data logger.

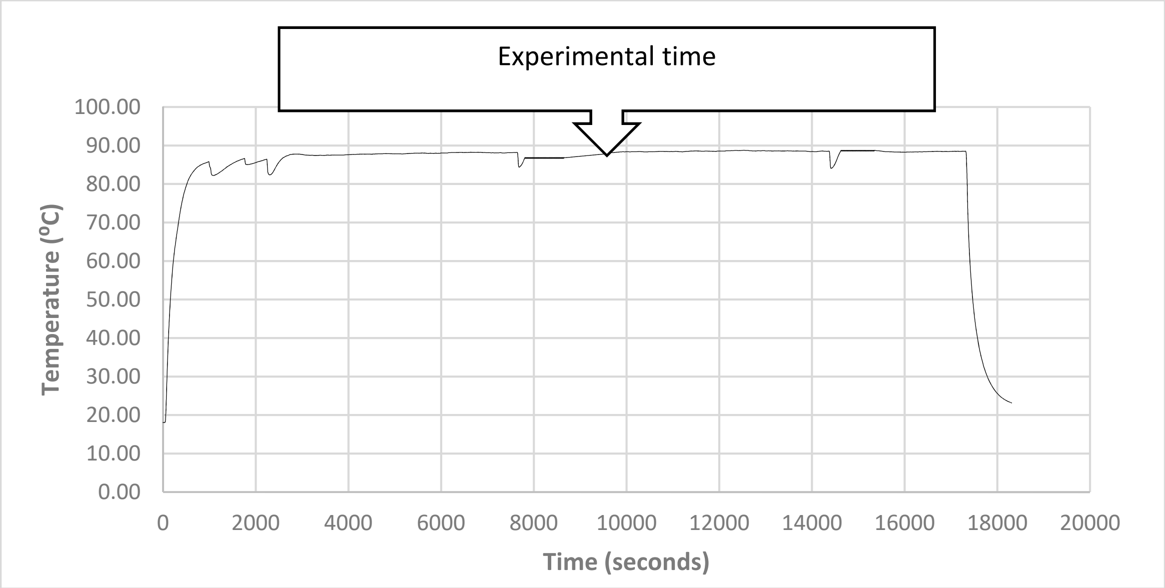


**Figure S4**: Temperature profile for the log-reduction assay at 85 °C for up to 240 min measured with a Tinytag data logger.

**Table S1:** Visual assessment of *Mycobacterium tuberculosis* (*M. tb.*) on 7HllP agar plates after different dry heat treatment regimens, and subsequent incubation for 3 weeks at 37 °C.

| Temperature and duration | Inoculum type | Replicate 1 | Replicate 2 | Replicate 3 | Replicate 4 | Replicate 5 | Replicate 6 |
|---|---|---|---|---|---|---|---|
| PC | *M. tb.* only | 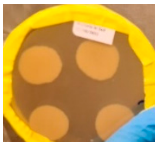 | 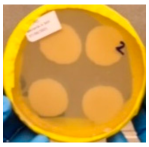 | 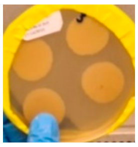 | 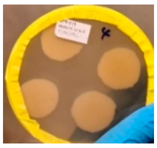 | 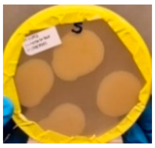 | 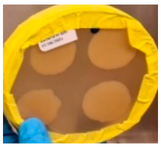 |
| PC | Soil load | 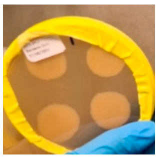 | 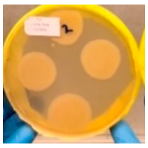 | 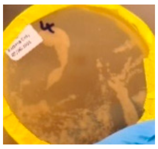 | 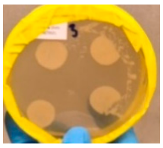 | 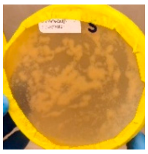 | 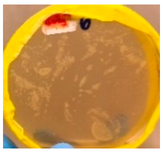 |
| 75 °C 30 min | *M. tb.* only | 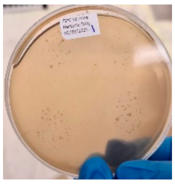 | 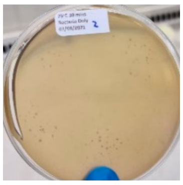 | 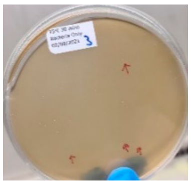 | 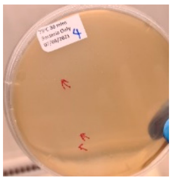 | No growth | No growth |
| 75 °C 60 min | *M. tb.* only | 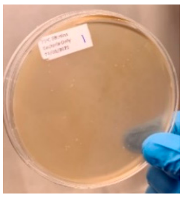 | 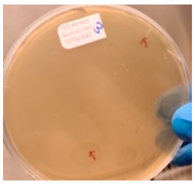 | No growth | No growth | No growth | No growth |
| 85 °C 30 min | *M. tb.* only | No growth | No growth | 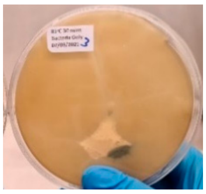 *Contaminant* | No growth | No growth | No growth |
| 85 °C 30 min | Soil load | No growth | No growth | No growth | No growth | 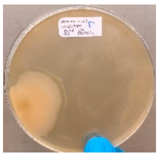 *Contaminant* | No growth |

PC, positive controls kept at room temperature for 10 min 18-22 °C).

Plates labelled as "Contaminant" are individual replicates that were contaminated with rapidly growing non-mycobacterial bacteria.

Growth of less than 10 colonies per plate is indicated by a red arrow, since the size of bacterial colonies was ≈1 mm and difficult to see in a photograph otherwise.
